# A TP53 Related Immune Prognostic Model for the Prediction of Clinical Outcomes and Therapeutic Responses in Lung Adenocarcinoma

**DOI:** 10.3389/fimmu.2022.876355

**Published:** 2022-06-28

**Authors:** Xiaonan Zhang, Simin Min, Yifan Yang, Dushan Ding, Qicai Li, Saisai Liu, Tao Tao, Ming Zhang, Baiqing Li, Shidi Zhao, Rongjing Ge, Fan Yang, Yan Li, Xiaoyu He, Xiaoxiao Ma, Lian Wang, Tianyu Wu, Tao Wang, Guowen Wang

**Affiliations:** ^1^ Department of Pathophysiology, Bengbu Medical College, Bengbu, China; ^2^ Bengbu Medical College Key Laboratory of Cardiovascular and Cerebrovascular Diseases, Bengbu, China; ^3^ Department of Thoracic Surgery, The First Affiliated Hospital of Bengbu Medical College, Bengbu, China; ^4^ Department of Immunology, Bengbu Medical College, Bengbu, China; ^5^ Department of Preventive Medicine, Bengbu Medical College, Bengbu, China; ^6^ College of Life and Health Sciences, Northeastern University, Shenyang, China

**Keywords:** LUAD, TP53, immunotherapy, immune prognostic model, TIM

## Abstract

TP53 is the most frequently mutated gene in lung adenocarcinoma (LUAD). The tumor immune microenvironment (TIM) is considered a vital factor that influences tumor progression and survival rate. The influence of TP53 mutation on TIM in LUAD has not been fully studied. Here we systematically investigated the relationship and potential mechanisms between TP53 mutation status and immune response in LUAD. We constructed an immune prognostic model (IPM) using immune associated genes, which were expressed differentially between the TP53 mutant and wild type LUAD patients. We discovered that TP53 mutations were significantly associated with 5 immune related biological processes. Thirty-six immune genes were expressed differentially between TP53 mutant and wild type LUAD patients. An IPM was constructed using 3 immune genes to differentiate the prognostic survival in LUAD. The high-risk LUAD group displayed significantly higher proportions of dendritic cell resting, T cell CD4 memory resting and mast cell resting, and significantly low proportions of dendritic cell activated, T cell CD4 memory activated, and mast cell activated. Moreover, IPM was found to be an independent clinical feature and can be used to predict immunotherapy responses. In summary, we constructed and validated an IPM using 3 immune related genes, which provides a better understanding of the mechanism from an immunological perspectives.

## Introduction

Lung cancer (LC) is the leading cause of cancer death worldwide, which leads to approximately 1.6 million deaths per year (1, 2). Non-small cell lung cancer (NSCLC) accounts for about 85% of LC patients (3). Lung adenocarcinoma (LUAD) is the most prevalent subtype of NSCLC and comprises approximately 40% of all LC cases (4). Most LUAD patients are caused by chronic cigarette smoking, which accounts for more than 80% of patients all over the world (5).

The most common genetic mutated genes in LUAD are TP53 (mutated in 46% of cases), KRAS (32%), and EGFR (27%) (1). Despite the achievements in uncovering the molecular mechanisms of this disease, the treatment of LUAD patients is still very limited. Currently, surgery, chemotherapy, radiotherapy, and targeted therapy are commonly used to treat patients with LUAD. However, in spite of the great improvement in clinical diagnosis and therapeutic treatments, the 5-year overall survival (OS) rate of LUAD patients is still <20% due to the advanced stage, late diagnosis, high recurrence rate, and poor survival (1).

In the last few years, immunotherapy has been developed widely as an additional treatment for anti-PD1 agents (1). This suggests the significance of the tumor immune microenvironment (TIM) on the clinical outcomes of patients with LUAD. TIM has been regarded as a critical factor influencing tumor progression (6). Tumor immune cells are the most dominant cell types that could be used as potential biomarkers for tumor prognostic prediction.

TP53, the most frequently mutated gene in LUAD, exerts a key role in maintaining genomic stability, and losing the function of TP53 could induce chromosomal instability and aneuploid cell proliferation. Interestingly, more and more studies have shown that TP53 mutations are related to different immune responses (7, 8).

Despite the fact that the pathogenetic effects of TP53 mutation on the clinical prognosis of LUAD patients have been extensively studied extensively, the effect of TP53 mutations on TIM in LUAD has not been fully studied. Therefore, here we systematically investigated the relationship and potential mechanisms between TP53 mutation status and immune response in LUAD.

## Materials and Methods

### Data Collection

Somatic mutations, gene expression profiles, and corresponding clinical information of LUAD patents were retrieved from the TCGA database (9). Patients with somatic mutations and expression were obtained, respectively. We collected their clinical information, including age, gender, race, stage, number of smoking packs per year, primary therapy outcome, and survival date.

The gene expression profiles and corresponding clinical information of GSE68465, GSE72094, and GSE78220 were downloaded from the GEO database. Of those, GSE68465 based on the GPL96 platform includes the expression profiling clinical information of 442 LUAD patients (10). GSE72094, based on the GPL15048 platform, has 442 LUAD patients with gene expression, driver, and tumor suppressor mutations, and clinical overall survival (OS) (11). GSE78220 based on the GPL11154 platform contains somatic mutations and transcriptomes of pre-treatment melanoma (12).

From August 2009 to December 2020, 100 patients diagnosed as primary LUAD at the First Affiliated Hospital of Bengbu Medical College participated in this study (Bengbu cohort). Two blinded pathologists evaluated the HE stained slides of each patient. Eventually, ten LUAD samples were chosen to investigate the protein expression of interested genes.

### Gene Set Enrichment Analysis (GSEA)

To study the differences in the immune genes and immunological pathways between TP53 wildtype and mutant patients in the TCGA LUAD cohort, GSEA (13) was carried out by GSEA software (v4.0) using the c5.bp.v7.1.symbols.gmt gene set from the Molecular Signatures Database (14). The cutoff was set at P <0.05.

### Differentially Expressed Gene (DEG) Analysis

The trimmed mean of M value (TMM) was used to normalize the downloaded data by using the edge R package (v3.24.3) in R. Deseq2 (15) was used to perform DEG analysis using raw counts calculated by HTSeq (14). DEGs were determined with a cutoff of an adjust P-value of less than 0.01 and |log fold change| greater than 0.5.

### Construction of the Immune-Associated Prognostic Model (IPM)

Among the LUAD patients having transcription data and TP53 mutation information, LUAD subjects with survival information were referred for additional analysis. The expression patterns of the DEGs from LUAD participants with survival information were evaluated by univariate Cox regression analysis. The prognostic significance of the DEGs for OS was established by univariate Cox regression analysis. In this study, genes were classified as significant at P <0.05. For highly correlated genes, the univariate Cox regression model cannot be utilized directly; consequently, least absolute shrinkage and selection operator (LASSO) with L1-penalty (16), which is a common approach for constructing interpretable prediction rules that can manage the collinearity issue, was employed. Among the immune genes that were notable in the univariate Cox regression analysis, critical immune genes were chosen using the LASSO approach. In this strategy, a sub-selection of immune genes implicated in LUAD patient prognosis was identified by the shrinking of the regression coefficient by the implementation of a penalty proportionate to their magnitude. Finally, a very limited number of indications with a weight of nonzero persisted, and most of the possible indicators were decreased to zero. Thus, LASSO-penalized Cox regression was performed to further minimize the number of immune genes. In this research, we subsampled the dataset 1,000 times and identified the immune genes that were repeated >900 times. LASSO Cox analysis was done by utilizing the glmnet R package (17). Finally, an immune-related prognostic model was created by employing the regression coefficients generated from multivariate Cox regression analysis to multiply the expression level of each immune gene. X-tile software (v3.6.1) was employed to find the optimal cutoff for LUAD patients categorized as low-risk and high-risk. The log-rank test and Kaplan–Meier survival analysis were performed to examine the prediction performance of the prognostic model.

### Estimation of Immune Cell Proportion

CIERSORT is a computational framework to quantify accurately the relative proportions of different immune cells using a gene expression admixture (18). We employed CIBERSORT using the LM22 signature to evaluate the proportions of 22 human hematopoietic cells for each patient.

### Functional Enrichment Analysis

The DAVID (v6.8) (9, 19) and the KO-based annotation system (KOBAS, v3.0) were used to perform GO (20, 21) and KEGG (22–25) enrichment analyses to explore the biological implications. GO analysis can reveal the underlying regulatory pattern among the gene expression profiles. KEGG pathway enrichment analysis is a frequently used method to determine the primary pathway of genes.

### Construction of Nomogram Using Prognostic Related Features

Among the LUAD patients with survival information, patients with complete clinical information, namely, age, gender, race, stage, number of smoking packs per year, primary therapy outcome, and survival date, were used for univariate and multivariable Cox regression to explore the prognostic related features in clinical. The nomogram using prognostic related features was built to predict 1-, 3-, and 5-year OS rates of LUAD patients *via* the rms R package (v5.1.3). A receiver operating characteristic (ROC) curve and a calibration curve were constructed to evaluate the accuracy of the nomogram. A concordance index (C index) was employed to examine the discrimination of the nomogram by the 1,000 resamples bootstrap method (26).

### Immunohistochemistry (IHC)

The tumor tissue from the LUAD samples was fixed with 4% paraformaldehyde, paraffin-embedded, and cut into 4-um sections. The paraffine sections were incubated with anti-EXO1, anti-COCH, and anti-CD40LG primary antibodies at 4°C overnight, according to the standard protocols. Each section was evaluated by two blinded pathologists independently. The intensity scale was defined as follows: 3—strong, 2—moderate, 1—weak, and 0—negative.

### Subcutaneous Tumorigenesis in Nude Mice

All experimental procedures were approved by the Animal Care and Use Committee of the Bengbu Medical College. The healthy mice were raised under pathogen-free conditions and provided with standard drinking water and food. All surgical procedures were performed under aseptic conditions. Tumor growth was measured every 4 days, and tumor volume was calculated as follows: tumor volume = 0.5 ∗ the longest diameter ∗ the shortest diameter^2^. Mice were randomly grouped into control, PF-4708671 50 mg/kg, and PF-4708671 75 mg/kg groups. The drug was injected intraperitoneally. At the end of the experiments, the mice were euthanized by cervical dislocation, and the tumor was resected and weighed.

### Statistical Analysis

R software was employed to perform the statistical analysis in this study. A *P*-value of less than 0.05 was deemed to be significant statistically for Kaplan–Meier survival analysis using the log-rank test with 95% CI. Continuous variables were analyzed using a t-test for normal distribution. The Kruskal–Wallis (KW) test was used for the association between gene expression and different anti-PDL1 responses of LUAD patients. ROC analysis was carried out by using the pROC R package.

## Results

### Relationships Between TP53 Mutations and Immune Response

**Figure 1:**
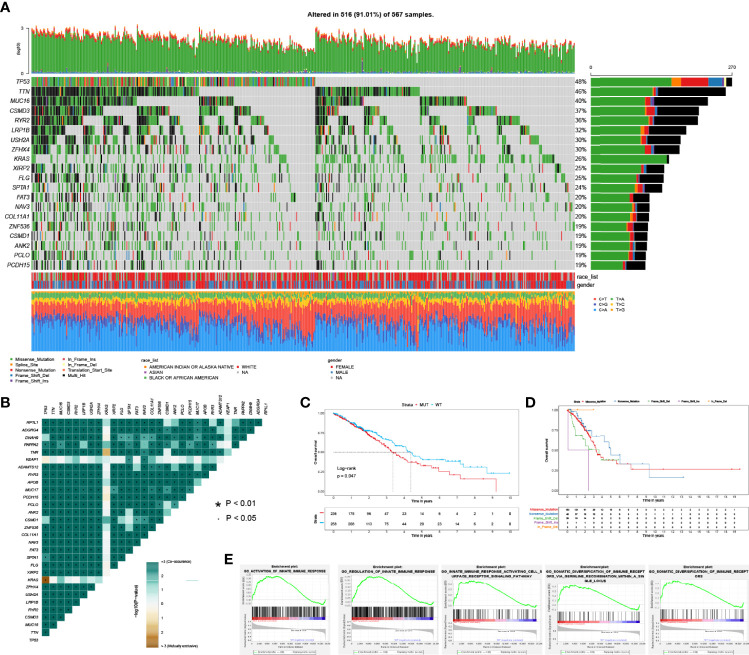
The spectrum of mutated genes and enrichment analysis based on TP53 status in LUAD patients. **(A)** Frequently mutated genes of LUAD patient in TCGA. **(B)** Co-occurrence and mutually exclusive analysis of mutated genes in LUAD. **(C)** Overall survival between TP53 mutant and wild type groups. **(D)** Overall survival stratified by different mutation types of TP53. **(E)** Significantly enriched biological processes between TP53 mutant and wild type comparison.

TP53 was the most frequently mutated gene in LUAD (270 cases, 48%, **Figure 1A**). Interestingly, we found that TP53 mutations were mutually exclusive with KRAS mutations while they co-occurred with mutations of other genes, like TTN and MUC16 (**Figure 1B**). Moreover, TP53 mutations were significantly related to poor OS in LUAD (**Figure 1C**). There was no difference among different mutation types of TP53 regarding OS in LUAD (**Figure 1D**). Next, we performed GSEA analysis of LUAD patients without (n = 258) and with (n = 236) TP53 mutations. The results demonstrated that TP53 wild type patients were significantly enriched in 5 immune-associated biological processes: regulation of innate immune response; activation of innate immune response; innate immune response activation of the cell surface receptor signaling pathway, etc. (**Figure 1E**; **Table S1**).

### Identification of Differentially Expressed Immune Genes Associated With TP53 Mutations

To discover the differentially expressed immune genes related to TP53 mutations in LUAD patients, we employed the DESeq2 package to perform differential expression analysis. Of the 5 immune processes, 36 genes were differentially expressed between TP53 mutant and wild type LUAD patients, with the cutoff of FDR <0.05 and |log2FC| >0.5 (**Table S2**).

### Construction of IPM and Evaluation of its Predictive Value in Different LUAD Cohorts

**Figure 2:**
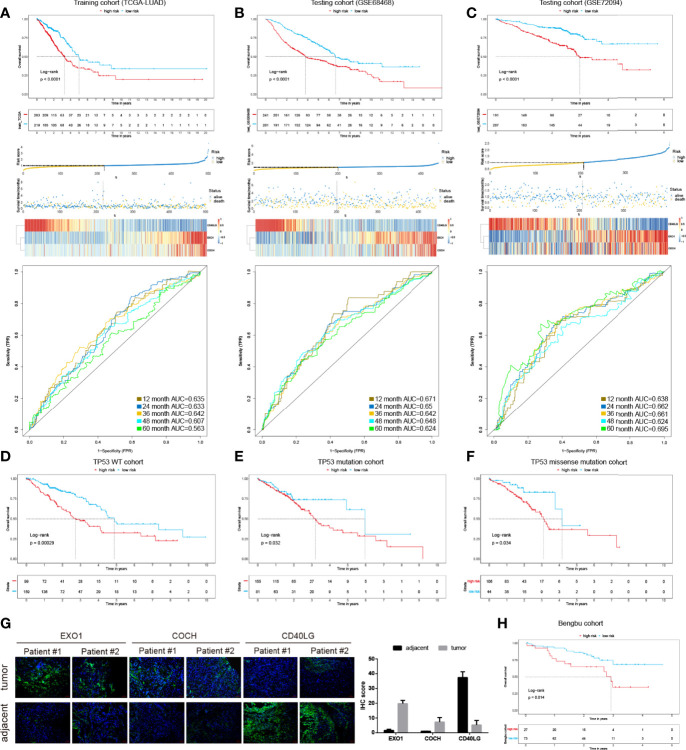
The development and validation of the immune prognostic model (IPM). KM survival risk table, and ROC cures of the IPM in TCGA LUAD **(A)**, GSE68468 **(B)**, and GSE72094 **(C)** cohorts. KM survival curves of overall survival according to TP53 wild type groups **(D)**, TP53 mutation **(E)**, and TP53 missense mutation subgroups **(F)**. **(G)** IHC of EXO1, COCH, and CD40LG in tumor and adjacent samples. **(H)** KM survival curves in the Benbu cohort.

To identify prognostic related immune genes, we first used univariate Cox regression and discovered five OS-associated genes out of the 36 DEGs. Next, we employed the LASSO approach to identify the genes with the most prognostic value. Finally, three genes (EXO1, COCH, and CD40LG) were selected (**Table S3**). Then, we built the risk score model by multiplying the LASSO regression coefficients to the normalized gene expression of each immune gene (riskscore = EXO1 ∗ 0.14 − CD40LG ∗ 0.199 + COCH ∗ 0.082). The patients were divided into the high- or low-risk groups based on their risk scores. As shown in **Figure 2A**, the high-risk group displayed worse OS than the low-risk group in the TCGA training cohorts (P <0.05). The AUC of the prognostic model for OS was 0.635 at 1 year, 0.633 at 2 years, 0.642 at 3 years, 0.607 at 4 years, and 0.563 at 5 years, respectively.

We also evaluated the IPM performance in the GEO LUAD cohorts (GSE68468 and GSE72094) to validate the performance of the IPM. Consistent with the findings of the TCGA LUAD cohort, the high-risk group displayed significantly poorer OS than the low-risk group (**Figures 2B, C**). The AUC values in different years were greater than 0.5 in the GSE68468 and GSE72094 cohorts. These findings demonstrated the applicability and robustness of the identified IPM in different cohorts. Classification analysis demonstrated that IPM was significantly associated with OS in wild type, mutated TP53, and TP53 missense-mutated groups (**Figures 2D–F**). Moreover, we validated the gene expression of these 3 genes in two replicates experimentally (**Figure 2G**). Also, we validated the IPM in our cohort and observed consistent results with other LUAD cohorts mentioned above (**Figure 2H**).

### Immune Landscape Between the High- and Low-Risk LUAD Patients

**Figure 3:**
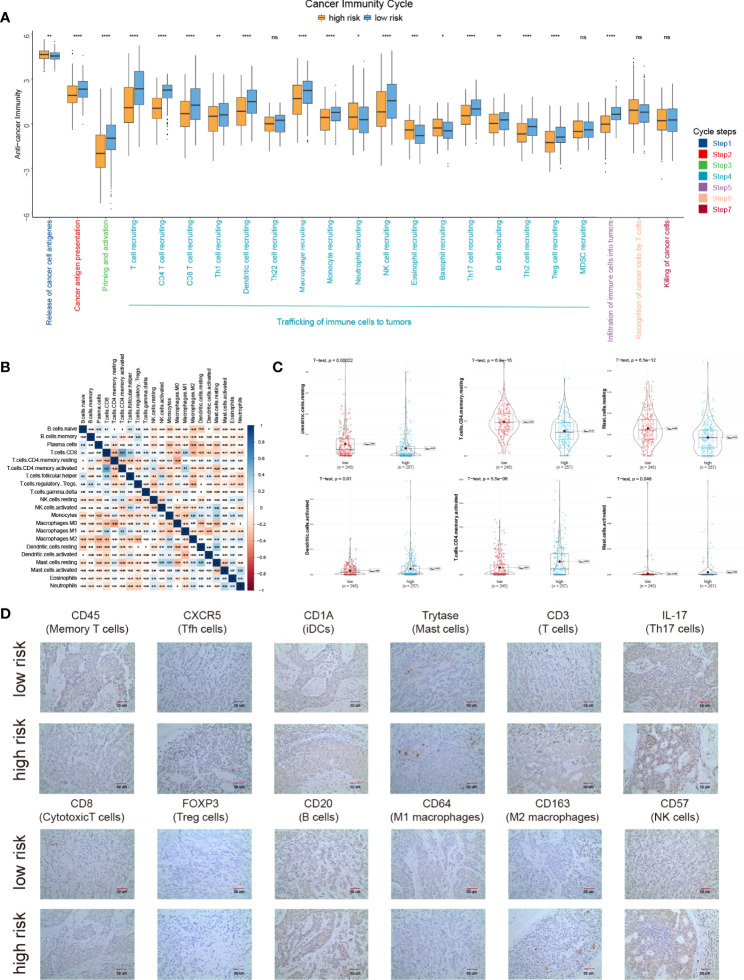
The landscape of immune infiltration in high- and low-risk LUAD patients. **(A)** Cancer immunity cycles between low and high risk groups. **(B)** Correlation of the proportions of 22 different immune cell. **(C)** Significantly different immune cells between low- and high-risk groups. **(D)** Proteins expression of different immune cells between low- and high-risk groups. *P < 0.05, **P < 0.01, ***P < 0.001, ****P < 0.0001, ns P > 0.05.

The cancer immunity cycle is a series of 7 steps used to describe how the immune system recognizes and kills cancer cells. Here, we compared the anti-cancer immunity in different cancer immunity cycles between IPM high- and low-risk groups. Interestingly, we found that the low-risk group displayed higher anti-cancer immunity in most cancer immunity steps, especially in steps 2 to 5 (**Figure 3A**). This conformed to the clinical outcomes of different groups. Moreover, using CIBERSORT with the LM22 signature, we found that the fractions of different immune cells in tumor were moderately or weakly correlated (**Figure 3B**). The high-risk LUAD group displayed higher proportions of some immune cells, namely, T cell CD4 memory resting, dendritic cell resting, and mast cell resting, and significantly lower proportions of dendritic cells, T cell CD4 memory, and mast cell activated (**Figure 3C**; **Figure S1**). Additionally, we validated these findings with experiments (**Figure 3D**).

**Figure 4:**
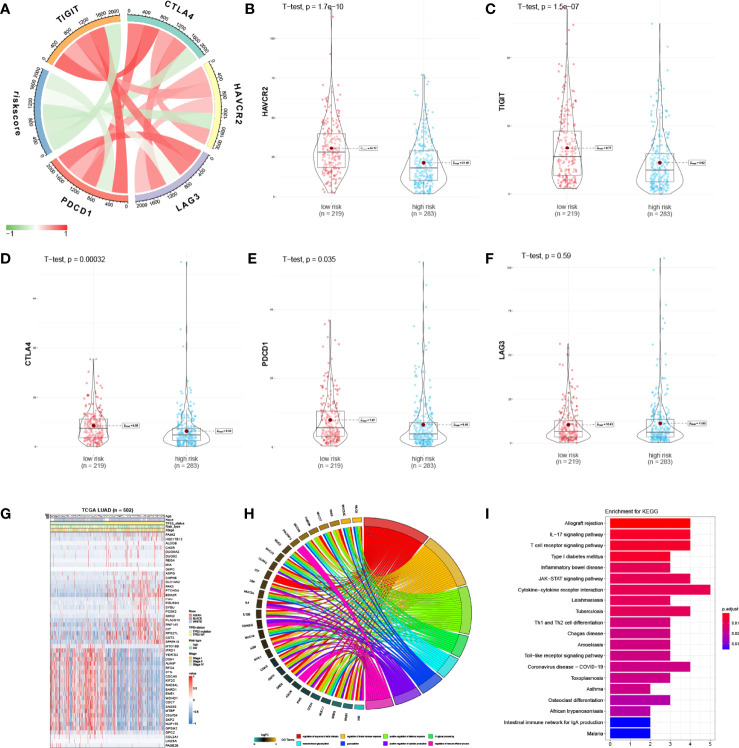
Enrichment analysis of IPM. **(A)** Correlation of the IPM risk score with immune checkpoints’ gene expression. **(B)** Violin plot of HAVCR2 between low- and high-risk groups. **(C)** Violin plot of TIGIT between low- and high-risk groups. **(D)** Violin plot of CTLA4 between low- and high-risk groups. **(E)** Violin plot of PDCD1 between low- and high-risk groups. **(F)** Violin plot of LAG3 between low- and high-risk groups. **(G)** Heatmap of significantly expressed immune genes between low* and high-risk groups. **(H)** Enriched biological processes of the immune genes. **(I)** Enriched KEGG pathways of the immune genes.

We next examined the correlations between IPM risk scores and the gene expression of immune checkpoint genes (TIGIT, CTLA4, HAVCAR2, LAG3, and PDCD1). Strikingly, we found that the risk scores were significantly related to the expression of HAVCAR2, TIGIT, CTLA4, and PDCD1 (**Figure 4A**; **Table S4**). Moreover, we discovered that the gene expression of these genes in the high-risk group was significantly higher than those in the low-risk group (**Figures 4B–F**), indicating that the poor prognosis of high-risk LUAD patients is partly due to the immune suppressive microenvironment.

### Altered Pathways Between Low- and High-Risk Group Patients

The differentially expressed immune genes between the low- and high-risk groups and the risk score correlated genes (correlation coefficient >0.5 and P <0.05) were deemed as risk-score-related genes. Fifty immune genes were selected (**Figure 4G**) and were used for GO and KEGG analyses. Based on the results, we found that these genes were significantly enriched in the immune responses and disease pathways of the immune system (**Figures 4H, I**; **Tables S5, S6**).

### The IPM was Independent of Traditional Clinical Features

**Figure 5:**
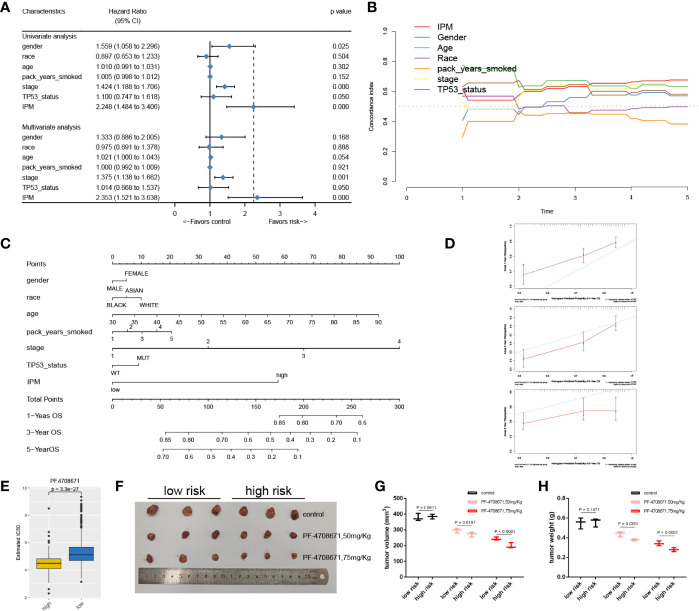
The connection between IPM and conventional clinical characteristics. **(A)** univariate and multivariate analyses of IPM, TP53 mutation status and clinical features. **(B)** C-index of IPM, TP53 mutation status, and clinical features. **(C)** Nomogram for predicting 1-, 3-, and 5-year OS for LUAD patients. **(D)** Calibration plot of the nomogram for predicting the probability of OS at 1, 3-, and 5-years. **(E)** IC50 between high and low risk groups. **(F)** Tumors were harvested and photographed from nude mice. **(G)** Tumor volume of low- and high-risk groups treated by PF4708671. **(H)** Tumor weight of low- and high-risk groups treated by PF4708671.

To study whether the IPM was independent of other clinical features in the TCGA LUAD cohort as for the prognostic value, we carried out univariate and multivariate Cox regression analyses. After adjusting for clinical features, including gender, age, race, pack year smoked, stage, and TP53 mutation status, the IPM was still an independent prognostic feature (**Figure 5A**). Moreover, we compared the C-index between the IPM and traditional clinical prognostic predictive features and found that the IPM had a higher mean C-index than others (**Figure 5B**). Taken together, these findings suggest that the IPM was independent of traditional clinical features and performed better than traditional clinical features for predicting prognosis in LUAD.

### Construction and Validation of the Nomogram Based on the IPM

To provide a quantitative method for clinicians to predict the clinical outcomes, we constructed a nomogram using the IPM and other clinical risk features (gender, race, age, pack years smoked, stage, and TP53 mutation status). We found that the IPM contributed more risk points than gender, race, pack years smoked, and TP53 mutation status, but less than age and stage (**Figure 5C**). The bias corrected line in the calibration plot was found to be close to the ideal curve, which indicated good agreement between the prediction and the observation (**Figure 5D**).

### High-Risk Group Was More Sensitive to PF4708671

Given that chemotherapy is the gold standard for cancer treatment, we evaluated the responsiveness of two risk subtypes to anticancer medicines, which are often employed as first-line therapies for cancer. We trained the predictive model using ridge regression on the GDSC cell line dataset and found that it had a satisfactory prediction accuracy as measured by 10-fold cross-validation. Drugs with P <0.01 were retained for further analysis (**Figure S2**). After that, we chose the most significant drug, PF4708671 (P = 3.3e−27) to validate the drug response *in vivo*, and the nude mice were classified by quantifying three IPM genes using qRT-PCR. PF4708671 can lead to apoptotic cell death (27). We compared the half maximal inhibitory concentration (IC50) between high- and low-risk groups. Strikingly, we found that the patients in the high-risk group had a lower IC50 than those in the low-risk group (**Figure 5E**). Moreover, we observed that the tumor volume and tumor weight in the high-risk group significantly decreased after PF4708671 treatment compared with the low-risk group (**Figures 5F–H**).

### The IPM Score Predicted Immunotherapy Benefits

**Figure 6:**
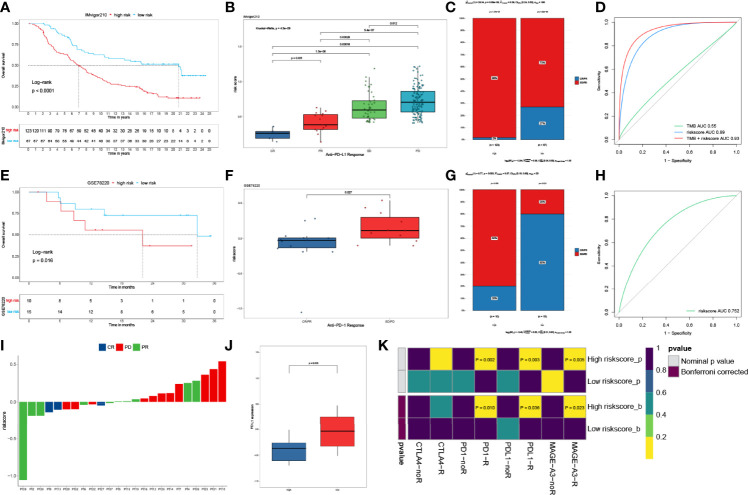
IPM predicts immunotherapeutic benefit. **(A)** KM curves for patients with low- and high-risk scores in the IMvigor210 cohort. **(B)** Risk score distribution with different anti-PDL1 clinical responses in the IMvigor210 cohort. **(C)** Relative proportion of clinical response to anti-PDL1 immunotherapy in low- and high-risk groups in the IMvigor210 cohort (PD, progressive disease; SD, stable disease; PR, partial response; CR, complete response). **(D)** ROC curves of TMB, risk score and combination of TMB and risk score in IMvifor210 cohort. **(E)** KM curves for patients in low- and high-risk groups in the GSE78220 cohort. **(F)** Distribution of risk scores with different anti-PDL1 clinical responses in the GSE78220 cohort. **(G)** Relative proportion of clinical response to anti-PDL1 immunotherapy in low and high risk groups in the GSE78220 cohort. **(H)** ROC curves of risk score in the GSE78220 cohort. **(I)** The IPM risk score of each patient. **(J)** Gene expression levels of PR-partial response between low- and high-risk groups. **(K)** Putative immune therapy response of low- and high-risk groups.

We next explored the prognostic value of the IPM for immune checkpoint therapy using IMvifor210 and GSE78220 cohorts. Patents with high IPM scores had significantly worse prognoses than those with low IPM scores in both the IMvifor210 and GSE78220 cohorts (**Figures 6A, E**). Patients with low IPM scores may benefit more from immune checkpoint therapy (**Figures 6B, C, F, G**). The tumor mutation burden (TMB), which is significantly related to immunotherapy efficiency, was also assessed using the ROC. Nevertheless, we found that TMB alone did not have predictive value compared with the IPM risk score. But, a combination of TMB and IPM risk score improved the predictive power compared with using TMB or IPM alone (**Figures 6D, H**). The risk score for each sample is shown in **Figure 6I**. Moreover, we compared the expression profiles of the two IPM risk groups with another cohort, which contained 47 melanoma patients who responded to immunotherapies. Strikingly, we discovered that patients in the high-risk group were more sensitive to anti-PD1/PDL1 therapy (**Figures 6J, K**).

## Discussion

TP53 has been identified as an indicator of anti-PD1 immunotherapy in LC (7). However, how the TP53 mutations regulate the LUAD immunophenotype and thus affect the prognosis of LUAD has not been studied. Therefore, it is important to study the immune-associated influence of the TP53 mutation status. Moreover, it is necessary to build a useful immune-associated prognostic model to predict the immune status.

In this study, we studied the influence of the TP53 mutations on the immune processes in LUAD. *Via* GSEA analysis, we observed that TP53 wild type LUAD displayed a stronger immune phenotype than those with TP53 mutations significantly. Next, we identified the immune associated genes influenced by TP53 mutations and constructed the 3 gene-based IPM, which could predict the clinical outcomes of LUAD patients. These genes (EXO1, COCH, and CD40LG) that constitute IPM might be used as individual targets.

EXO1 has been reported to be involved in mammalian non-homologous end joining and leads to drug resistance in ovarian cancer (28, 29). Moreover, high expression of EXO1 has been stated to be related to poor OS of breast cancer and hepatocellular carcinoma (30, 31). Similarly, Zhou et al. reported that EXO1 is a potential prognostic marker and correlates with tumor infiltrating immune cells in LUAD (32). COCH is a highly conserved gene and the loss of function of COCH mutations leads to autosomal recessive non-syndromic hearing loss (33). Also, COCH is regulated at the time of embryo implantation by leukemic inhibitory factor in the uterus (34). CD40LG, expressed on the T-cell surface, can regulate B-cell functions by engaging CD40 on the B-cell surface (35–37). CD40LG has been identified as a prognostic biomarker that regulates TME *via* immune processes in breast cancer (38). In this study, we found the high expression of EXO1, COCH, and CD40LG was related to an unfavorable clinical outcome in LUAD patients.

Furthermore, we established that the IPM maintained a significant independent predictive component once clinical features were changed. This finding implies that the local immune state may be able to enhance the conventional characteristics of accurate prediction. As a result, we offer a complete evaluation that incorporates our IPM and other clinical characteristics. For 1-, 3-, and 5-year OS, the calibration curve demonstrated acceptable agreement between observed and anticipated values. The primary benefit of our model is that it provides a complementary viewpoint on individual tumors and produces a patient-specific scoring system; hence, our nomogram could be a useful tool for physicians in the future.

According to the cancer immunoediting theory, during cancer growth in immunological-competent hosts, fewer immunogenic cancer cells are picked (immune selection) and immunosuppressive networks are formed (immune escape) to avoid anti-tumor immune responses. Thus, clinically significant cancers have multiple immunosuppressive mechanisms, including an increase in various immunosuppressive cells (e.g., Treg cells and tumor-associated macrophages), an increase in the expression of various immunosuppressive molecules (e.g., CTLA-4), and a decrease in the expression of cancer antigens, resulting in the inability of CD8^+^ T cells to recognize cancer cells. By inhibiting the action of immunosuppressive cells and processes, it is possible to activate anti-tumor immune responses. The purpose of this study was to compare the immunological mechanisms of low- and high-risk patients as well as the potential use of cancer immunotherapy to increase the anti-tumor immune response. The findings suggested that the proposed technique had promising clinical effectiveness. Additionally, we compared immune checkpoint expression (CTLA-4, PD-1, and TIM-3) between the low- and high-risk groups. CTLA-4, PD-1, and TIM-3 expression was substantially greater in high-risk HCC patients than in low-risk patients (P <0.05). Prior research established that CD4 memory resting T cells can be further differentiated and confer a variety of functions, including inhibiting CD8^+^ T cell activation and NK cell killing, suppressing harmful immune responses to self- and foreign antigens, and assisting CD8+ T cells in tumor rejection. Notably, Tregs exhibited a variety of immunological checkpoints, including PD-1 and CTLA-4. Ipilimumab, an anti-CTLA-4 antibody, disrupts associations between antigen-presenting cells (APCs) and regulatory T cells (Tregs). Anti-CTLA-4 antibody analyses in mouse models revealed that their antitumor efficacy is based on the depletion of CTLA-4+ Treg cells in tumors *via* antibody-dependent cellular cytotoxicity (ADCC), as the loss of crystallizable fragment (Fc) function of anti-CTLA-4 mAbs completely eliminates their antitumor effects. Thus, in our model, the risk score was consistent with the ability of tumor-infiltrating immune cells to assess the expression of immune checkpoints, implying that the high-risk group’s poor prognosis may be due to a more immunosuppressive environment and immune checkpoint expression than in the low-risk group, which promoted LUAD growth, development, invasion, and angiogenesis and contributed to a poor prognosis. Additionally, these findings show that patients at a higher risk would benefit more from immune checkpoint inhibitors than those at a lower risk, resulting in a better prognosis.

In summary, we fully investigated the mechanisms of TP53 mutation status and their effects on the immune response in LUAD patients. We identified and validated an IPM using 3 immune-related genes, which provides a better understanding of the mechanism from an immunological perspective.

## Data Availability Statement

Publicly available datasets were analyzed in this study. This data can be found here: All data used in this work can be acquired from the Gene-Expression Omnibus (GEO; https://www.ncbi.nlm.nih.gov/geo/) under the accession number GSE68468 and GSE72094, and the GDC portal (https://portal.gdc.cancer.gov/).

## Ethics Statement

The studies involving human participants were reviewed and approved by the Medical Ethics Committee of the First Affiliated Hospital of Bengbu Medical College. The patients/participants provided their written informed consent to participate in this study. The animal study was reviewed and approved by the Medical Ethics Committee of the First Affiliated Hospital of Bengbu Medical College.

## Author Contributions

XZ, TaW, and GW conceived and designed this work. XZ, SM, YY, and TaW performed the majority of the experiments, analyzed the data. XZ and SM developed the methodology. XZ, DD, TT, SL, MZ, and BL collected data. XZ, QL, SZ, RG, FY, and YL provided administrative, technical, or material support. XZ, GW, YY, XH and XM wrote and edited the manuscript. XZ, GW, DD, LW, and TiW reviewed the manuscript. All authors listed have made a substantial, direct, and intellectual contribution to the work and approved it for publication.

## Funding

This work was supported by the National Natural Science Foundation of China 81673791 and the Education fund item of Anhui province under Grant KJ2020A0588.

## Conflict of Interest

The authors declare that the research was conducted in the absence of any commercial or financial relationships that could be construed as a potential conflict of interest.

## Publisher’s Note

All claims expressed in this article are solely those of the authors and do not necessarily represent those of their affiliated organizations, or those of the publisher, the editors and the reviewers. Any product that may be evaluated in this article, or claim that may be made by its manufacturer, is not guaranteed or endorsed by the publisher.
