# Supplementary material for: A TP53 Related Immune Prognostic Model for the Prediction of Clinical Outcomes and Therapeutic Responses in Lung Adenocarcinoma
Source: Front Immunol. 2022 Jun 28;13:876355. doi: 10.3389/fimmu.2022.876355 (PMC9275777; doi:10.3389/fimmu.2022.876355)
Supplement: Supplementary file 1 [file DataSheet_1.pdf]

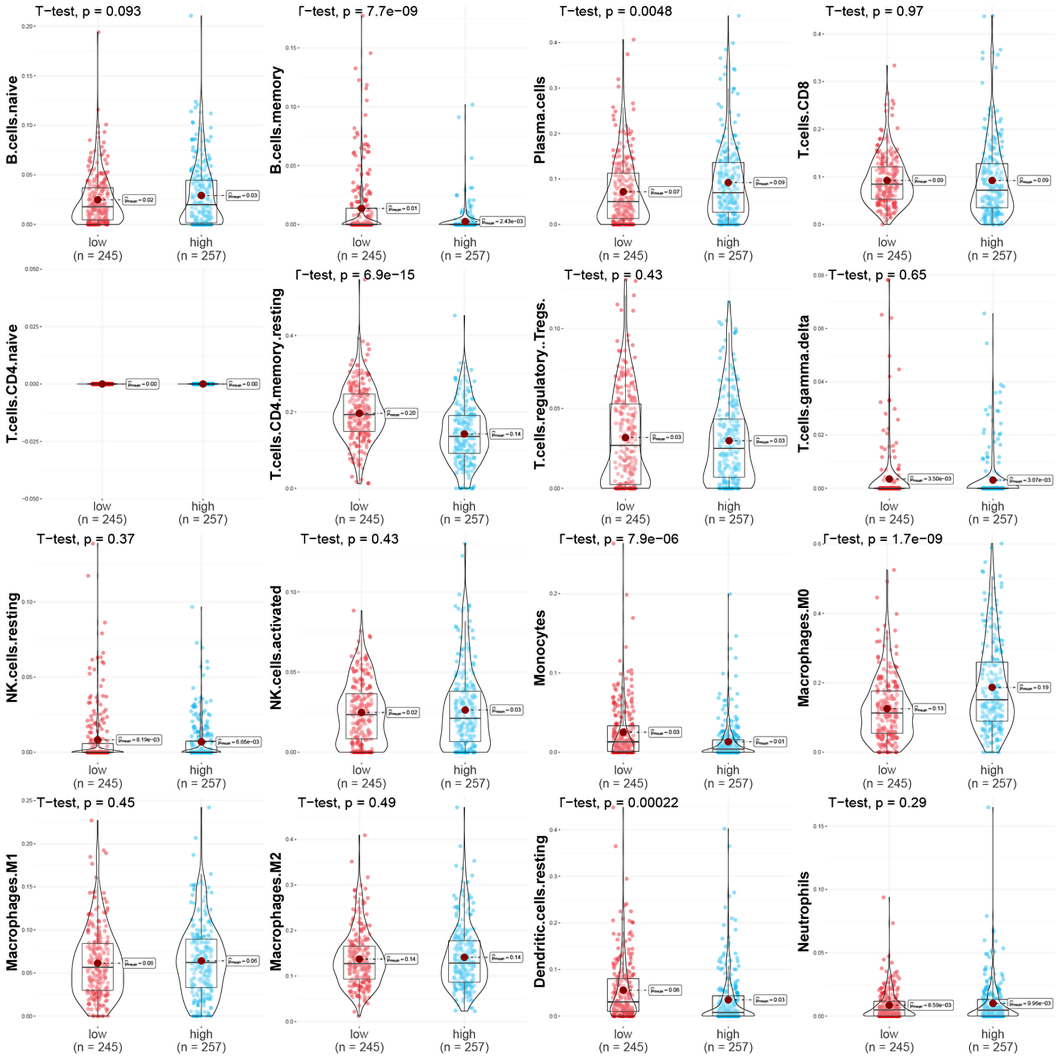

Fig. S1. different immune cells between low and high risk groups.

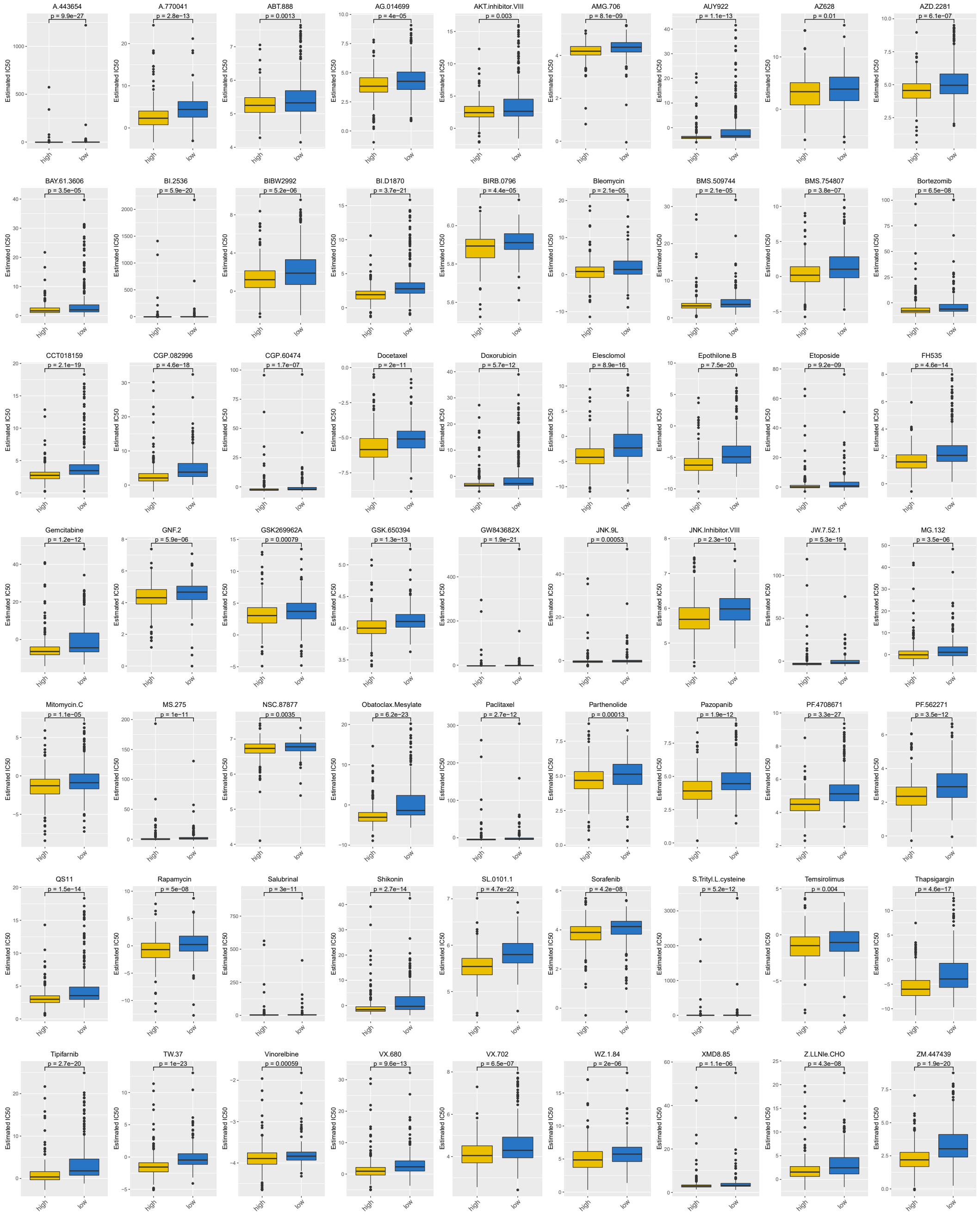

Fig. S2. IC50 of significant drugs between high and low risk groups.
